# *Umbellaceae* fam. nov. (*Hymenochaetales*, *Basidiomycota*) for *Umbellus sinensis* gen. et sp. nov. and Three New Combinations

**DOI:** 10.3390/jof10010022

**Published:** 2023-12-28

**Authors:** Xue-Wei Wang, Li-Wei Zhou

**Affiliations:** 1 State Key Laboratory of Mycology, Institute of Microbiology, Chinese Academy of Sciences, Beijing 100101, China; xuewei_wang1995@im.ac.cn; 2 University of Chinese Academy of Sciences, Beijing 100049, China

**Keywords:** *Basidiomycota*, corticioid fungi, macrofungi, six new taxa, *Umbellus*

## Abstract

*Hymenochaetales*, belonging to *Agaricomycetes*, *Basidiomycota*, comprises most polypores and corticioid fungi and, also, a few agarics. The latest taxonomic framework accepts 14 families in this order. When further exploring species diversity of *Hymenochaetales*, two corticioid specimens collected from China producing cystidia with large umbrella-shaped crystalline heads attracted our attention. This kind of cystidia was reported only in three unsequenced species, viz. *Tubulicrinis corneri*, *T. hamatus* and *T. umbraculus*, which were accepted in *Tubulicrinaceae*, *Hymenochaetales*. The current multilocus-based phylogeny supports that the two Chinese specimens formed an independent lineage from *Tubulicrinaceae* as well as the additional 13 families and all sampled genera in *Hymenochaetales*. Therefore, a monotypic family, *Umbellaceae,* is newly described with the new genus *Umbellus* as the type genus to represent this lineage. The two Chinese specimens are newly described as *U. sinensis*, which differs from *T. corneri*, *T. hamatus,* and *T. umbraculus* in a combination of a smooth to grandinioid hymenophoral surface, not flattened, broadly ellipsoid basidiospores with a tiny apiculus, and growth on angiosperm wood. Due to the presence of the unique cystidia, the three species of *Tubulicrinis*, even though they lack available molecular sequences, are transferred to *Umbellus* as *U. corneri*, *U. hamatus,* and *U. umbraculus*. Hereafter, all known species with large umbrella-shaped crystalline-headed cystidia are in a single genus. In summary, the current study provides a supplement to the latest taxonomic framework of *Hymenochaetales* and will help to further explore species diversity and the evolution of this fungal order.

## 1. Introduction

*Hymenochaetales* was described as a monotypic order to accommodate *Hymenochaetaceae* by Frey et al. [1]. This fungal order, belonging to *Agaricomycetes*, *Basidiomycota* [2], is globally distributed in the forest ecosystem, and for now comprises 14 families and 83 genera, of which 19 genera have no certain position at the family level [3]. Most of the species in *Hymenochaetales* are polypores and corticioid fungi, whereas certain species, like those in the genera *Blasiphalia*, *Contumyces,* and *Rickenella*, are agarics. In addition to the morphological diversity, various trophic modes, including saprotrophs, parasites, and symbiotes (with both tree and moss), also exist in *Hymenochaetales*. More importantly, some polypores of *Hymenochaetales*, like those in the genera *Sanghuangporus* and *Phylloporia* among others, are highly valuable medicinal fungi [4,5]. Therefore, species in *Hymenochaetales* can be important in the forest ecosystem and for economic development as strategic biological resources [6].

While the species diversity has been well explored all over the world [7,8,9,10,11,12,13,14,15,16,17], the systematics of *Hymenochaetales* at the family level were poorly established. The families recorded in several papers were even contradictory. This phenomenon was mainly due to the samplings in phylogenetic analyses with a biased emphasis on target fungal groups [18,19] and was also caused by the unreliable phylogenetic analyses inferred only from one or two ribosomal loci [18,20]. This was the case until, recently, Wang et al. [3] systematically summarized the taxonomic background and updated the taxonomic framework of *Hymenochaetales* via multilocus phylogenetic analyses on the basis of the most comprehensive samplings. This update provides a crucial basis for further exploring species diversity and the taxonomic positions of species in *Hymenochaetales*.

The cystidium is a sterile structure but possesses unique importance in fungal taxonomy, especially for corticioid fungi that normally have simple morphological traits. Among various kinds of cystidia, large umbrella-shaped crystalline-headed cystidia are rarely present and are known only in three species, viz. *Tubulicrinis corneri*, *T. hamatus,* and *T. umbraculus* [21,22,23]. *Tubulicrinis*, typified by *T. glebulosus*, was placed in *Tubulicrinaceae*, *Hymenochaetales* for the first time by Larsson [20]. This opinion is accepted by Wang et al. [3], treating *Tubulicrinaceae* as a monotypic family. Unfortunately, the molecular sequences are unavailable from *T. corneri*, *T. hamatus,* and *T. umbraculus*. Therefore, the phylogenetic relationships among these three species and other species in *Tubulicrinis* are unknown.

When examining two corticioid specimens collected in China, umbrella-shaped crystalline-headed cystidia were observed. To identify these two specimens at a species level and determine their taxonomic position at higher ranks, careful morphological examinations and phylogenetic analyses were performed. In addition to the unique cystidia, other key taxonomic morphological characters of these two specimens were different from *T. corneri*, *T. hamatus,* and *T. umbraculus*. Moreover, these two specimens occupied an independent lineage from *Tubulicrinaceae* as well as the additional 13 families and all sampled genera in *Hymenochaetales*. Therefore, these two specimens are described as a new species belonging to a new genus in a new monotypic family. In addition, *T. corneri*, *T. hamatus,* and *T. umbraculus* are transferred to the new genus.

## 2. Materials and Methods

### 2.1. Morphological Examination

The two studied specimens were deposited at the Fungarium, Institute of Microbiology, Chinese Academy of Sciences (HMAS), Beijing, China.

Macromorphological characters were examined with the aid of a Leica M125 stereomicroscope (Wetzlar, Germany) at magnifications of up to 100×. Special color terms followed Petersen [24]. Micromorphological characters were examined with an Olympus BX43 light microscope (Tokyo, Japan) at magnifications of up to 1000×, following Wang et al. [25]. Specimen sections were separately mounted in Cotton Blue, Melzer’s reagent, and 5% potassium hydroxide. All measurements were made from the sections mounted in Cotton Blue. When presenting the variation in basidiospore sizes, 5% of the measurements were excluded from each end of the range and are given in parentheses. Drawings were made with the aid of a drawing tube. The following abbreviations are used in the descriptions: L = mean basidiospore length (arithmetic average of all measured basidiospores), W = mean basidiospore width (arithmetic average of all measured basidiospores), Q = variation in the L/W ratios between the studied specimens, and (n = a/b) = number of basidiospores (a) measured from given number of specimens (b).

The detailed structure of cystidia was examined with a Hitachi SU8000 scanning election microscope (Tokyo, Japan). The sections of basidiomes were sprayed with gold and platinum using Leica EM ACE600 (Wetzlar, Germany).

### 2.2. Molecular Sequencing

Crude DNA was extracted from basidiomes of dry specimens as templates for subsequent PCR amplifications using FH Plant DNA Kit (Beijing Demeter Biotech Co., Ltd., Beijing, China) according to the manufacturer’s instructions. The nrSSU, ITS, nrLSU, mtSSU, and RNA polymerase II second largest subunit (*RPB2*) regions were amplified using the selected primer pairs PNS1/NS41 [26], ITS1F/ITS4 [27], LR0R/LR7 [28], MS1/MS2 [29], and fRPB2-5F/fRPB2-7cR [30] and bRPB2-6F/bRPB2-7.1R [31], respectively. The PCR procedures for nrSSU and mtSSU regions were as follows: initial denaturation at 94 °C for 3 min, followed by 34 cycles at 94 °C for 40 s, 55 °C for 45 s, and 72 °C for 1 min and a final extension at 72 °C for 10 min. For ITS region, they were as follows: initial denaturation at 95 °C for 3 min, followed by 34 cycles at 94 °C for 40 s, 57.2 °C for 45 s, and 72 °C for 1 min and a final extension at 72 °C for 10 min. For nrLSU region they were as follows: initial denaturation at 94 °C for 1 min, followed by 34 cycles at 94 °C for 30 s, 47.2 °C for 1 min, and 72 °C for 1.5 min and a final extension at 72 °C for 10 min. And, for *RPB2* region, they were as follows: initial denaturation at 94 °C for 2 min, followed by 9 cycles at 94 °C for 40 s, 60 °C for 40 s, and 72 °C for 2 min and 36 cycles at 94 °C for 45 s, 55 °C for 1.5 min, and 72 °C for 2 min, and a final extension at 72 °C for 10 min. With the same primers used in PCR amplifications, the PCR products were sequenced at the Beijing Genomics Institute, Beijing, China, and the resulting new sequences were deposited in GenBank (https://www.ncbi.nlm.nih.gov/genbank/; accessed on 7 July 2023; Table 1).

### 2.3. Phylogenetic Analyses

In addition to the newly generated sequences for this study, additional related sequences, mainly following Wang et al. [3], were also integrated in phylogenetic analyses (Table 1).

The dataset with a combination of nrSSU, ITS, nrLSU, mtSSU, and *RPB2* regions was used to explore the phylogenetic position of the newly sequenced specimens in *Hymenochaetales*. Within *Hymenochaetales*, all sequenced species with uncertain taxonomic positions at the family level and selected representatives of all 14 previously accepted families were included. Meanwhile, two species from *Polyporales*, viz. *Fomitopsis pinicola* and *Grifola frondosa,* were also included, and two species from *Thelephorales*, viz. *Boletopsis leucomelaena* and *Thelephora ganbajun,* were selected as outgroup taxa [3].

Each of the five regions was separately aligned using MAFFT v.7.110 [32] under the “G-INS-i” option [33]. Due to the crucial role of gaps for delimiting taxa at the higher taxonomic level [34], they were reserved as the fifth character for all five regions. Then, the alignments of the five regions were concatenated as a single alignment (File S1). The best-fit evolutionary models of the concatenated alignment and each single-region alignment were estimated using jModelTest v.2.1.10 [35,36] under Akaike information criterion. Maximum Likelihood (ML) and Bayesian Inference (BI) algorithms were utilized for phylogenetic analyses of the concatenated alignment, and ML algorithm was utilized for phylogenetic analyses of each single-region alignment. The ML algorithm was conducted using raxmlGUI v.8.2.12 [37] and the bootstrap (BS) replicates were calculated under the auto FC option [38]. The BI algorithm was conducted using MrBayes v.3.2.7 [39]. Two independent runs, with each run including four chains and starting from random trees, were employed. Trees were sampled every 1000th generation. Of the sampled trees, the first 25% were removed while the other 75% were retained for constructing a 50% majority consensus tree and calculating Bayesian posterior probabilities (BPPs). Chain convergence was judged using Tracer v.1.7.1 [40] after discarding 25% of samples. The final phylogenetic tree was edited and visualized using tvBOT (https://www.chiplot.online/tvbot.html; accessed on 24 June 2023) [41].

## 3. Results

### 3.1. Molecular Phylogeny

In this study, nine sequences for the five regions used in phylogenetic analyses were newly generated from the two studied specimens, viz. LWZ 20190615-27 and LWZ 20190615-39, with the absence of the mtSSU sequence from the specimen LWZ 20190615-39 (Table 1).

The phylogenies generated from the five single-region alignments under the best-fit evolutionary model of GTR + I + G generally share rather similar topologies in their main lineages (Figures S1–S5). However, in each phylogeny, several species are not located in their supposed positions and the BS values are not high enough. This phenomenon indicates that a single region cannot well delimit the taxonomic relationship of Hymenochaetales. Therefore, multilocus-based phylogenetic analyses are necessary.

**Figure 1:**
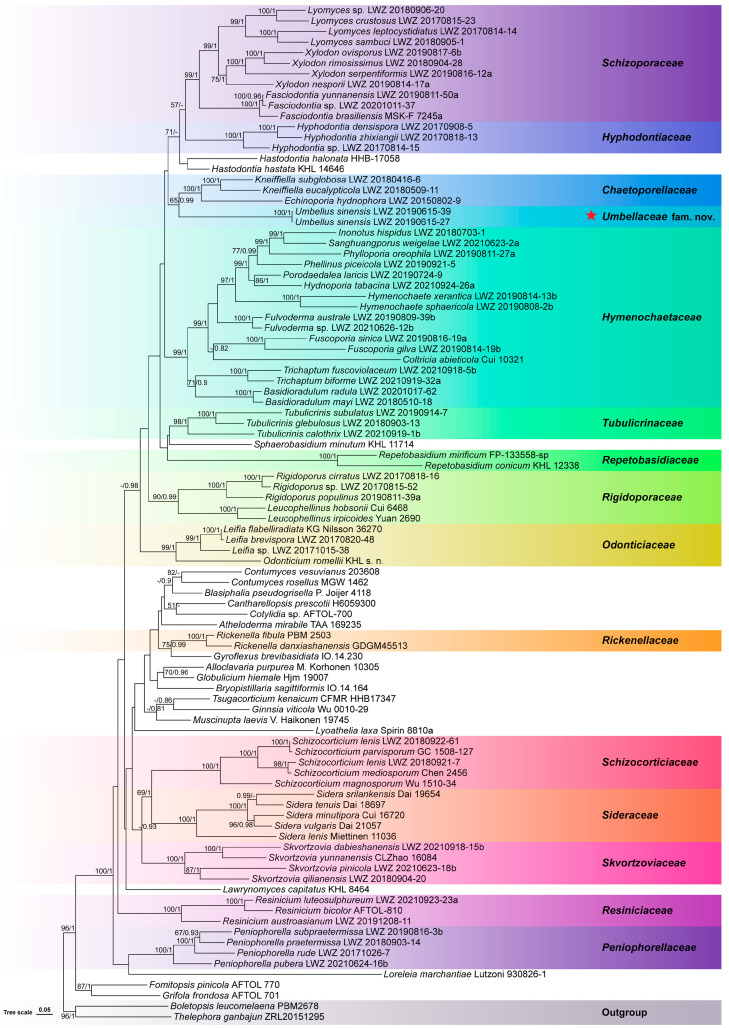
Phylogenetic position of *Umbellaceae* (marked with a red star) within *Hymenochaetales,* inferred from the combined dataset of nrSSU, ITS, nrLSU, mtSSU, and *RPB2* regions. The topology has been generated using the maximum likelihood algorithm. The maximum likelihood bootstrap values and the Bayesian posterior probability values above 50% and 0.8, respectively are shown at the nodes. *Boletopsis leucomelaena* and *Thelephora ganbajun* from *Thelephorales* have been selected as outgroup taxa.

The combined dataset of nrSSU, ITS, nrLSU, mtSSU, and *RPB2* regions from 96 collections generated a concatenated alignment of 5190 characters with GTR + I + G as the best-fit evolutionary model. In the ML algorithm, the BS search stopped after 150 replicates. In the BI algorithm, after 25 million generations with an average standard deviation of split frequencies of 0.008948, all chains converged, which was indicated by the effective sample sizes of all parameters being above 6600 and all potential scale reduction factors being equal to 1.000. ML and BI algorithms generated similar topologies in main lineages, and thus, the topology generated by the ML algorithm is presented along with BS values and BPPs above 50% and 0.8, respectively at the nodes (Figure 1). In this phylogeny, the monophyly of *Hymenochaetales* receives full statistical support, and within *Hymenochaetales*, the two newly sequenced specimens collected from Guangdong, China, group together as an independent lineage (BS = 100%, BPP = 1) from all sampled families and genera. Taking the unique characters of the two specimens into consideration together, we describe them as members of a new species of a new genus in a new family.

### 3.2. Taxonomy

***Umbellaceae*** Xue W. Wang & L.W. Zhou, **fam. nov.**

**MycoBank: MB 851425**

**Etymology**: *Umbellaceae* (Lat.), referring to the type genus *Umbellus*.

**Diagnosis**: Distinguished from other families of *Hymenochaetales* by capitate cystidia with large umbrella-shaped crystalline heads.

**Type genus**: *Umbellus* Xue W. Wang & L.W. Zhou.

**Type species**: *Umbellus sinensis* Xue W. Wang & L.W. Zhou.

**Description**: Basidiomes annual, adnate and resupinate. Hymenophore smooth to grandinioid or odontioid to hydnoid, white to cream; margin thinning out, arachnoid, concolorous or paler than subiculum. Hyphal system monomitic; generative hyphae with clamp connections. Cystidia dimorphic: (1) arising from subhymenium and more or less enclosed in the hymenium or strongly projecting for the greater part of their length, cylindrical, unevenly thick-walled with a narrow or wide lumen, rooted at the base, gradually tapering, broadly rounded at the apex and covered by a large umbrella-shaped crystalline head; (2) originating laterally on subicular hyphae, with the same morphology as those arising from subhymenium but smaller in size and stalk slightly thick-walled. Basidia subclavate to clavate-cylindrical, barrel-shaped or suburniform, with a basal clamp connection and four sterigmata. Basidiospores oblong-ellipsoid or broadly ellipsoid, hyaline, smooth, thin-walled, indextrinoid, inamyloid, acyanophilous.

**Notes**: Morphologically, the monotypic family *Umbellaceae* resembles *Chaetoporellaceae*, *Hyphodontiaceae*, and *Schizoporaceae* due to its resupinate basidiomes and light-colored hymenophoral surface, but differs in having capitate cystidia with large umbrella-shaped crystalline heads [3].

***Umbellus*** Xue W. Wang & L.W. Zhou, **gen. nov.**

**MycoBank: MB 851426**

**Etymology**: *Umbellus* (Lat.), referring to the large umbrella-shaped crystalline head of cystidia.

**Diagnosis**: Distinguished by capitate cystidia with a large umbrella-shaped crystalline head.

**Type**: *Umbellus sinensis* Xue W. Wang & L.W. Zhou.

**Description**: Basidiomes annual, adnate and resupinate. Hymenophore smooth to grandinioid or odontioid to hydnoid, white to cream; margin thinning out, arachnoid, concolorous or paler than subiculum. Hyphal system monomitic; generative hyphae with clamp connections. Cystidia dimorphic: (1) arising from subhymenium and more or less enclosed in the hymenium or strongly projecting for the greater part of their length, cylindrical, unevenly thick-walled with a narrow or wide lumen, rooted at the base, gradually tapering, broadly rounded at the apex and covered by a large umbrella-shaped crystalline head; (2) originating laterally on subicular hyphae with the same morphology as those arising from subhymenium but smaller in size and stalk slightly thick-walled. Basidia subclavate to clavate-cylindrical, barrel-shaped or suburniform, with a basal clamp connection and four sterigmata. Basidiospores oblong-ellipsoid or broadly ellipsoid, hyaline, smooth, thin-walled, indextrinoid, inamyloid, acyanophilous.

**Notes**: The two studied specimens, described as *Umbellus sinensis* below, are distinguished by the capitate cystidia with umbrella-shaped crystalline heads. Previously, three species of *Tubulicrinis*, viz. *T. corneri*, *T. hamatus*, and *T. umbraculus*, were reported to have this kind of cystidium [21,22,23]. In the current phylogeny, the lineage formed by the two studied specimens is separated from *Tubulicrinis* (Figure 1). Therefore, they cannot be placed in *Tubulicrinis*. In addition, while *T. corneri* was originally described in *Tubulicrinis* [21], the basionyms of *T. hamatus* and *T. umbraculus* belong to *Peniophora* [22,23]. *Peniophora* is a genus accepted in *Russulales* and thus cannot accommodate the two studied specimens. Therefore, a new genus, *Umbellus*, is introduced to accommodate species with the unique cystidia. Previously, the three species with the large umbrella-shaped crystalline-headed cystidia were all placed in the same genus, *Tubulicrinis*. For now, the fourth species with this kind of cystidium has been phylogenetically proven in a new genus, *Umbellus*. Therefore, although the molecular sequences of *T. corneri*, *T. hamatus*, and *T. umbraculus* are unavailable for phylogenetic analyses, these three species are transferred to *Umbellus* on the basis of their unique cystidia that hereafter are only known in this genus.

***Umbellus corneri*** (Jülich) Xue W. Wang & L.W. Zhou, **comb. nov.**

**MycoBank: MB 851427**

**Basionym:***Tubulicrinis corneri* Jülich, Persoonia 10(3): 332 (1979).

***Umbellus hamatus*** (H.S. Jacks. & Donk) Xue W. Wang & L.W. Zhou, **comb. nov.**

**MycoBank: MB 851428**

**Basionym:** *Peniophora hamata* H.S. Jacks., Canadian Journal of Research, Section C 26: 133 (1948).

≡ *Tubulicrinis hamatus* (H.S. Jacks.) Donk [as *‘*hamata*’*], Fungus, Wageningen 26 (1–4): 14 (1956).

**Figure 2:**
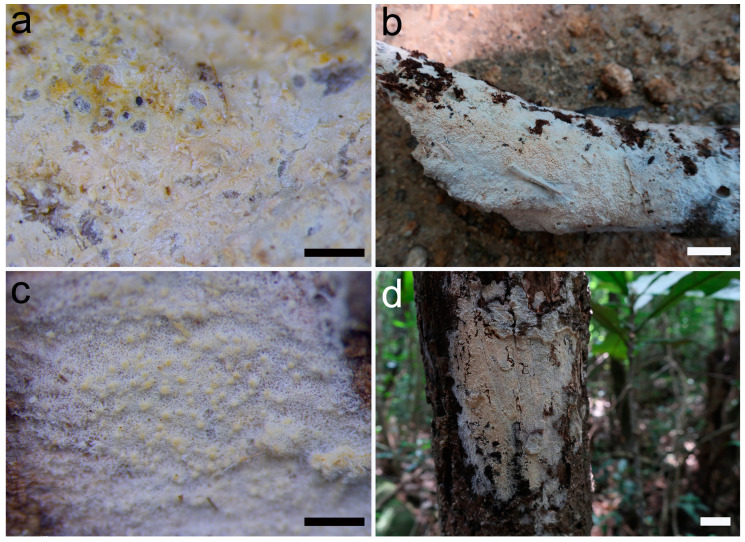
Basidiomes of *Umbellus sinensis*. (**a**,**b**) LWZ 20190615-27 (holotype). (**c**,**d**) LWZ 20190615-39 (paratype). Scale bars: (**a**,**c**) = 0.1 mm, (**b**,**d**) = 1 cm.

**Figure 3:**
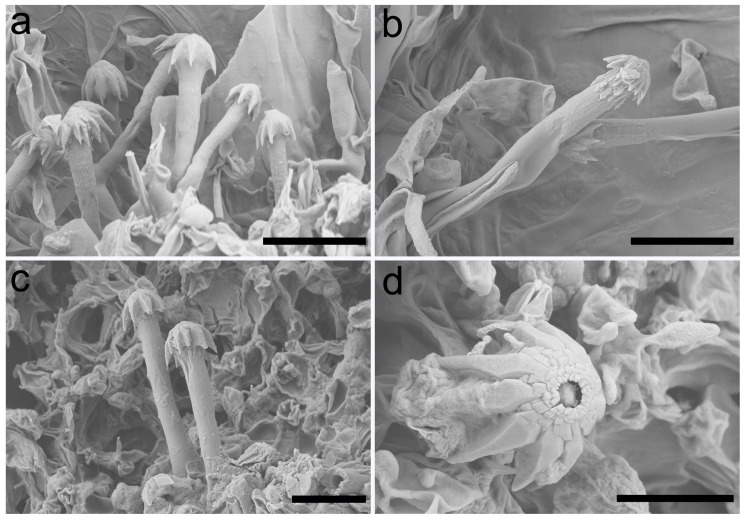
Scanning electron micrograph of cystidia of *Umbellus sinensis*. (**a**,**b**) LWZ 20190615-27 (holotype). (**c**,**d**) LWZ 20190615-39 (paratype). Scale bars: (**a**–**c**) = 10 μm, (**d**) = 5 μm.

**Figure 4:**
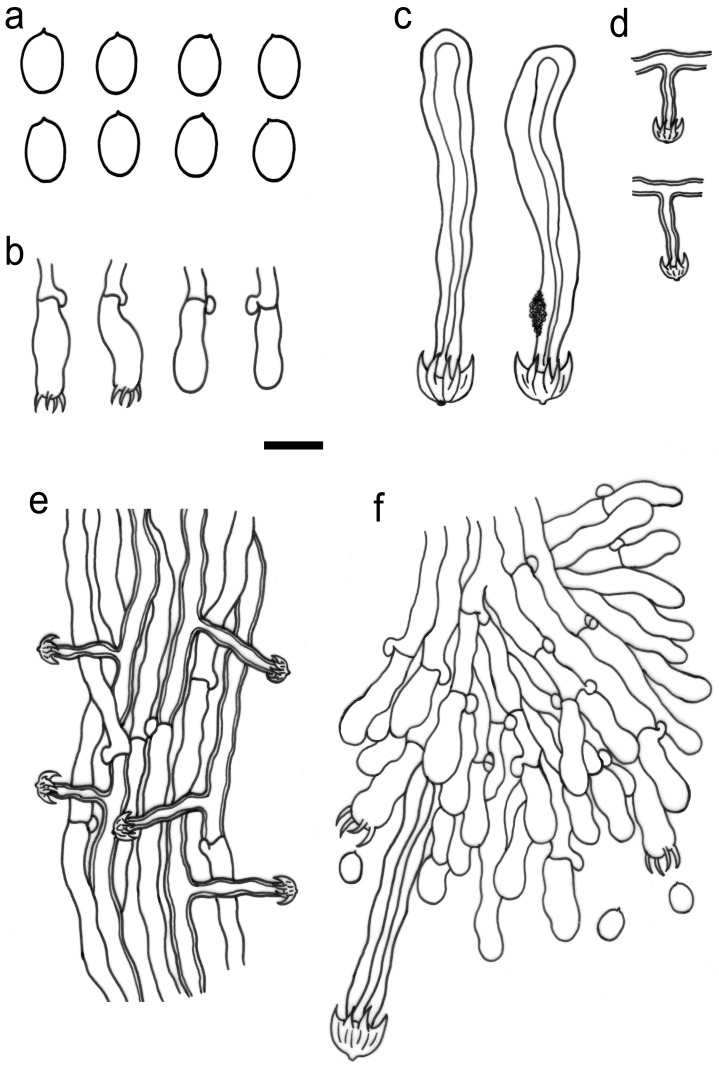
Microscopic structures of *Umbellus sinensis* (drawn from LWZ 20190615-27, holotype). (**a**) Basidiospores. (**b**) Basidia and basidioles. (**c**) Cystidia from the subhymenium. (**d**) Cystidia from subiculum. (**e**) Hyphae from subiculum. (**f**) A vertical section through hymenium. Scale bar: for (**a**) = 5 μm; for (**b**–**f**) = 10 μm.

***Umbellus sinensis*** Xue W. Wang & L.W. Zhou, **sp. nov.** (Figure 2, Figure 3 and Figure 4).

**MycoBank: MB 851429**

**Etymology**: *sinensis* (Lat.), referring to the type locality China.

**Diagnosis**: Distinguished by smooth to grandinioid hymenophoral surface and not flattened, broadly ellipsoid basidiospores with a tiny apiculus.

**Type**: China, Guangdong Province, Huizhou, Boluo County, Xiangtoushan National Nature Reserve, on a fallen branch of an angiosperm, 15 June 2019, *Li-Wei Zhou*, LWZ 20190615-27 (Holotype in HMAS).

**Description**: Basidiomes annual, adnate and resupinate, easily cracked when dry. Hymenophore smooth to grandinioid, white to cream; margin thinning out, arachnoid, paler than subiculum. Hyphal system monomitic; generative hyphae with clamp connections. Subicular hyphae hyaline, branched, 4–5.5 µm in diam, thin- to slightly thick-walled. Subhymenial hyphae hyaline, thin-walled, 4–4.5 µm in diam. Cystidia dimorphic: (1) arising from subhymenium and strongly projecting out for the greater part of their length, cylindrical, 45–60 × 6.5–9.5 µm, unevenly thick-walled with a lumen up to 4 µm, with a narrow or wide lumen, rooted at the base, gradually tapering, broadly rounded at the apex and covered by a large umbrella-shaped crystalline head of up to 9 µm in diam, set with 10–12 deflexed and radiating ridges terminating in acute spines; (2) originating laterally on subicular hyphae with the same shape as those arising from subhymenium but smaller in size, 15–25 × 1.5–3.5 µm, with an umbrella-shaped crystalline head of 5–6 µm in diam, stalk slightly thick-walled. Basidia subclavate to barrel-shaped, with a basal clamp connection and four sterigmata, 15–17 × 5–7 µm. Basidiospores broadly ellipsoid, hyaline, smooth, thin-walled, inamyloid, indextrinoid, acyanophilous, 4.5–5(–5.1) × (3.2–)3.3–4.2(–4.3) µm, L = 4.80 µm, W = 3.47 µm, Q = 1.37–1.38 (n = 60/2).

**Additional specimen examined**: China, Guangdong Province, Huizhou, Boluo County, Xiangtoushan National Nature Reserve, on a fallen branch of an angiosperm, 15 June 2019, *Li-Wei Zhou*, LWZ 20190615-39 (Paratype in HMAS).

**Notes**. Compared with *Umbellus sinensis*, *U. corneri* differs in its odontioid to slightly hydnoid hymenophoral surface [21]; *U. hamatus* differs in the flattened on one side, larger basidiospores (5.5–7.5 × 4–4.5 µm) with a prominent lateral apiculus [23]; and *U. umbraculus* (transferred below) differs in obovate, flattened-on-one-side, longer basidiospores (5–6 µm in length) [22]. Noteworthily, *U. hamatus* is known only on coniferous wood [23], while the other three species of *Umbellus* grow on angiosperm wood [21,22].

***Umbellus umbraculus*** (G. Cunn.) Xue W. Wang & L.W. Zhou, **comb. nov.**

**MycoBank: MB 851430**

**Basionym**. *Peniophora umbracula* G. Cunn., Trans. Roy. Soc. N.Z. 83: 291 (1955).

≡ *Tubulicrinis umbraculus* (G. Cunn.) G. Cunn. [as *‘*umbracula*’*], Bull. N.Z. Dept. Sci. Industr. Res. 145: 142 (1963)

**A key to all four known species in *Umbellus***

1 Hymenophore odontioid or slightly hydnoid..........................................................*U. corneri*
1 Hymenophore smooth to grandinioid....................................................................................2
2 Basidiospores oblong-ellipsoid or obovate.......................................................*U. umbraculus*
2 Basidiospores broadly ellipsoid...............................................................................................3
3 Basidiospores flattened on one side, with a prominent lateral apiculus, 5.5–7.5 × 4–4.5 µm; on coniferous wood..............................................................................................*U. hamatus*
3 Basidiospores not flattened, with a tiny apiculus, 4.5–5 × 3.3–4.2 µm; on angiosperm wood...............................................................................................................................*U. sinensis*

## 4. Discussion

In this paper, the latest taxonomic framework of *Hymenochaetales,* proposed by Wang et al. [3], is supplemented by describing a new family, *Umbellaceae*. Although *Umbellaceae* is a monotypic family with the new genus *Umbellus* as the type genus, it occupies an independent phylogenetic position from all sampled families and genera in *Hymenochaetales* (Figure 1). Similarly, *Chaetoporellaceae* was also a monotypic family in *Hymenochaetales* when being reinstated; however, later study soon added one more genus to this family [3]. Therefore, it is reasonable to describe monotypic families to provide certain taxonomic positions at the family level for as many genera as possible, as if the phylogenetic evidence is solid. More importantly, the large umbrella-shaped crystalline-headed cystidia in *Umbellaceae* are unique in all fungal groups to our knowledge. In addition to the presence of unique cystidia, *Umbellaceae* also differs from *Tubulicrinaceae* in its lack of cylindrical, conical, multi-radicate cystidia with a capitate or subulate apex [3]. Therefore, the description of *Umbellaceae* is supported from both phylogenetic and morphological perspectives.

In the molecular era of fungal taxonomy, the generic position of a species can be easily determined using accurate molecular phylogenetic analyses [42]. Therefore, the transfer of a fungal species to another genus normally needs molecular evidence. However, in the current case, *Umbellus corneri*, *U. hamatus,* and *U. umbraculus* are rather old species, and we cannot sequence them now and in the foreseeable future. Moreover, the large umbrella-shaped crystalline heads of cystidia are an extremely unique morphological character in taxonomy, and could be tentatively considered to be synapomorphy. In addition to sharing the unique cystidia, *Umbellus corneri*, *U. hamatus,* and *U. umbraculus* also resemble *U. sinensis* in annual, adnate, resupinate basidiomes and a monomitic hyphal system with clamp-connected generative hyphae. Therefore, we transfer these species to *Umbellus* based on the morphological perspective, even though their molecular sequences are unavailable. Then, all known species with the unique cystidia are in a single genus.

After the description of *Umbellaceae* and *Umbellus*, a total of 15 families accommodating 65 genera are accepted in *Hymenochaetales* while an additional 19 genera in *Hymenochaetales* have no certain taxonomic positions at the family level [3]. The species diversity in most of these 19 genera has rarely been systematically explored with the aid of molecular evidence [43,44], and their morphological and phylogenetic relationships with the 15 known families have still not been resolved [3]. Therefore, it is too mature to assign them to any known or new families. Given above, the taxonomic framework of *Hymenochaetales* still needs to be further updated.

## 5. Conclusions

In summary, two Chinese corticioid specimens are newly described as *Umbellus sinensis*, and a new monotypic family *Umbellaceae,* typified by a new genus, *Umbellus,* is described to accommodate the new species in *Hymenochaetales*. Moreover, three combinations, viz. *Umbellus corneri*, *U. hamatus,* and *U. umbraculus,* are proposed for the species previously belonging to *Tubulicrinis*. The updated taxonomic framework of *Hymenochaetales* will help further explore species diversity and the evolution of this fungal order, which are the main aims of fungal taxonomy [45].

## Figures and Tables

**Table 1:** Information on taxa in *Agaricomycetes* used in phylogenetic analyses.

Order/Family	Species	Voucher	nrSSU	ITS	nrLSU	mt-SSU	*RPB2*
*Hymenochaetales*/*Chaetoporellaceae*	*Echinoporia hydnophora*	LWZ 20150802-9	ON063768	ON063639	ON063838	ON063707	
	*Kneiffiella eucalypticola*	LWZ 20180509-11		MT319410	MT319142	MT326421	
	*Kneiffiella subglobosa*	LWZ 20180416-6		MT319413	MT319145	MT326422	
-/*Hymenochaetaceae*	*Basidioradulum mayi*	LWZ 20180510-18	ON427363	MN017785	MN017792	ON463756	ON456070
	*Basidioradulum radula*	LWZ 20201017-62	ON063814	ON063684	ON063884	ON063747	ON100713
	*Coltricia abieticola*	Cui 10321	KY693761	KX364785	KX364804	KY693823	KX364876
	*Fulvoderma australe*	LWZ 20190809-39b	ON063771	ON063644	ON063843	ON063712	ON100686
	*Fulvoderma* sp.	LWZ 20210626-12b	ON063772	ON063646	ON063845	ON063714	ON100687
	*Fuscoporia gilva*	LWZ 20190814-19b	ON063775	ON063648	ON063848	ON063717	ON100734
	*Fuscoporia sinica*	LWZ 20190816-19a	ON063776	ON063649	ON427358	ON063719	ON100691
	*Hydnoporia tabacina*	LWZ 20210924-26a	ON063778	ON063651	ON063851	ON063720	ON100685
	*Hymenochaete sphaericola*	LWZ 20190808-2b	ON063783	ON063656	ON063855	ON063725	ON100700
	*Hymenochaete xerantica*	LWZ 20190814-13b	ON063784	ON063657	ON063856	ON063726	ON100699
	*Inonotus hispidus*	LWZ 20180703-1	ON063785	ON063659	ON063858	ON063727	ON100692
	*Phellinus piceicola*	LWZ 20190921-5	ON063790	ON063662	ON063862	ON063731	ON100695
	*Phylloporia oreophila*	LWZ 20190811-27a	ON063793	ON063665	ON063865	ON063733	ON100694
	*Porodaedalea laricis*	LWZ 20190724-9	ON063796	ON063668	ON063868	ON063735	ON100693
	*Sanghuangporus weigelae*	LWZ 20210623-2a	ON063799	ON063671	ON063870	ON063736	ON100697
	*Trichaptum biforme*	LWZ 20210919-32a	ON063832	ON063701	ON063901	ON063764	ON100730
	*Trichaptum fuscoviolaceum*	LWZ 20210918-5b	ON063834	ON063703	ON063903	ON063765	ON100732
-/*Hyphodontiaceae*	*Hyphodontia pachyspora*	LWZ 20170908-5		MT319426	MT319160	MT326431	MT326261
	*Hyphodontia zhixiangii*	LWZ 20170818-13		MT319420	MT319151	MT326424	MT326270
	*Hyphodontia* sp.	LWZ 20170814-15		MT319417	MT319148	MT326423	MT326269
-/*Odonticiaceae*	*Leifia brevispora*	LWZ 20170820-48	ON427367	MK343470	MK343474	ON463759	
	*Leifia flabelliradiata*	KG Nilsson 36270		DQ873635	DQ873635		
	*Leifia* sp.	LWZ 20171015-38	ON427368	ON427471	ON427354	ON463760	
	*Odonticium romellii*	KHL s. n.		DQ873639	DQ873639		
-/*Peniophorellaceae*	*Peniophorella praetermissa*	LWZ 20180903-14	ON063816	ON063686	ON063886	ON063749	ON100714
	*Peniophorella pubera*	LWZ 20210624-16b	ON063817	ON063687	ON063887	ON063750	ON100715
	*Peniophorella rude*	LWZ 20171026-7	ON063818	ON063688	ON063888	ON063751	ON100716
	*Peniophorella subpraetermissa*	LWZ 20190816-3b	ON063819	ON063689	ON063889	ON063752	ON100717
-/*Repetobasidiaceae*	*Repetobasidium conicum*	KHL 12338	DQ873646	DQ873647	DQ873647		
	*Repetobasidium mirificum*	FP-133558-sp	AY293155		AY293208	AY293243	
-/*Resiniciaceae*	*Resinicium austroasianum*	LWZ 20191208-11	ON063821	ON063691	ON063891	ON063753	ON100720
	*Resinicium bicolor*	AFTOL-810		DQ218310	AF393061		DQ457635
	*Resinicium friabile*	LWZ 20210923-23a	ON063822	ON063692	ON427362	ON063754	ON100719
-/*Rickenellaceae*	*Rickenella danxiashanensis*	GDGM45513	ON063823	MF326424		ON063755	ON100721
	*Rickenella fibula*	PBM 2503	MF319021	DQ241782	MF318953		DQ408115
-/*Rigidoporaceae*	*Leucophellinus hobsonii*	Cui 6468		KT203288	KT203309	KT203330	KT210365
	*Leucophellinus irpicoides*	Yuan 2690		KT203289	KT203310	KT203331	KT210366
	*Rigidoporus cirratus*	LWZ 20170818-16	ON427369	ON427472	ON427355	ON463761	ON456073
	*Rigidoporus populinus*	LWZ 20190811-39a	ON063803	ON063674	ON063874	ON063740	ON100702
	*Rigidoporus* sp.	LWZ 20170815-52	ON427370	ON427473	ON427356	ON463762	ON456074
-/*Schizocorticiaceae*	*Schizocorticium lenis*	LWZ 20180921-7	ON063827	ON063696	ON063896	ON063760	ON100726
	*Schizocorticium lenis*	LWZ 20180922-61	ON063829	ON063698	ON063898	ON063762	ON100728
	*Schizocorticium magnosporum*	Wu 1510-34		MK405351	MK405337		
	*Schizocorticium mediosporum*	Chen 2456		MK405359	MK405345		
	*Schizocorticium parvisporum*	GC 1508-127		MK405361	MK405347		
-/*Schizoporaceae*	*Fasciodontia brasiliensis*	MSK-F 7245a		MK575201	MK598734		
	*Fasciodontia yunnanensis*	LWZ 20190811-50a	ON063804	ON063675	ON427360	ON063741	ON100704
	*Fasciodontia* sp.	LWZ 20201011-37	ON063805	ON063676	ON427361	ON063742	ON100705
	*Lyomyces crustosus*	LWZ 20170815-23		MT319465	MT319201	MT326446	MT326275
	*Lyomyces leptocystidiatus*	LWZ 20170814-14		MT319429	MT319163	MT326512	MT326256
	*Lyomyces sambuci*	LWZ 20180905-1	ON063807	MT319444	MT319178	MT326438	MT326291
	*Lyomyces* sp.	LWZ 20180906-20	ON063808	ON063678	ON063878	ON063743	ON100707
	*Xylodon nesporii*	LWZ 20190814-17a	ON063809	ON063679	ON063879		ON100708
	*Xylodon ovisporus*	LWZ 20190817-6b	ON063810	ON063680	ON063880	ON063744	ON100709
	*Xylodon rimosissimus*	LWZ 20180904-28	ON063812	ON063682	ON063882	ON063745	ON100711
	*Xylodon serpentiformis*	LWZ 20190816-12a	ON063813	ON063683	ON063883	ON063746	ON100712
-/*Sideraceae*	*Sidera lenis*	Miettinen 11036		FN907914	FN907914		
	*Sidera minutipora*	Cui 16720	MW418078	MN621349	MN621348	MW424986	MW505865
	*Sidera srilankensis*	Dai 19654	MW418087	MN621344	MN621346	MW424989	MW505868
	*Sidera tenuis*	Dai 18697	MW418083	MK331865	MK331867	MW424988	MW505866
	*Sidera vulgaris*	Dai 21057	MW418090	MW198484	MW192009	MW424987	MW505869
-/*Skvortzoviaceae*	*Skvortzovia dabieshanensis*	LWZ 20210918-15b	ON063825	ON063694	ON063894	ON063757	ON100723
	*Skvortzovia pinicola*	LWZ 20210623-18b	ON063826	ON063695	ON063895	ON063758	ON100724
	*Skvortzovia qilianensis*	LWZ 20180904-20	ON063824	ON063693	ON063893	ON063756	ON100722
	*Skvortzovia yunnanensis*	CLZhao 16084		MW472754	MW473473	ON063759	ON100725
-/*Tubulicrinaceae*	*Tubulicrinis calothrix*	LWZ 20210919-1b	ON063835	ON063704	ON063904	ON063766	ON100733
	*Tubulicrinis glebulosus*	LWZ 20180903-13	ON063836	ON063705	ON063905		
	*Tubulicrinis subulatus*	LWZ 20190914-7	ON063837	ON063706	ON063906	ON063767	
-/*Umbellaceae*	*Umbellus sinensis*	LWZ 20190615-27	**OR240268**	**OR242616**	**OR236212**	**OR250300**	**OR242518**
	*Umbellus sinensis*	LWZ 20190615-39	**OR240269**	**OR242617**	**OR236213**		**OR242519**
-/*Incertae sedis*	*Alloclavaria purpurea*	M. Korhonen 10305	MF318986	MF319044	MF318895		
	*Atheloderma mirabile*	TAA 169235		DQ873592	DQ873592		
	*Blasiphalia pseudogrisella*	P. Joijer 4118	MF318989	MF319047	MF318898		
	*Bryopistillaria sagittiformis*	IO.14.164		MT232349	MT232303		MT242333
	*Cantharellopsis prescotii*	H6059300	MF318993	MF319051	MF318903		MF288855
	*Contumyces rosellus*	MGW 1462	MF319001	MF319059	MF318912		MF288859
	*Contumyces vesuvianus*	203608	MF319002		MF318913		MF288860
	*Cotylidia* sp.	AFTOL-700	AY705958	AY854079	AY629317	FJ436111	AY883422
	*Ginnsia viticola*	Wu 0010-29		MN123802	GQ470670		
	*Globulicium hiemale*	Hjm 19007		DQ873595	DQ873595		
	*Gyroflexus brevibasidiata*	IO.14.230		MT232351	MT232305		MT242335
	*Hastodontia halonata*	HHB-17058		MK575207	MK598738		
	*Hastodontia hastata*	KHL 14646		MH638232	MH638232		
	*Lawrynomyces capitatus*	KHL 8464		DQ677491	DQ677491		
	*Loreleia marchantiae*	Lutzoni 930826-1		U66432	U66432		
	*Lyoathelia laxa*	Spirin 8810a		MT305998	MT305998		
	*Muscinupta laevis*	V. Haikonen 19745	MF319004	MF319066	MF318921		MF288861
	*Sphaerobasidium minutum*	KHL 11714		DQ873652	DQ873653		
	*Tsugacorticium kenaicum*	CFMR HHB17347	JN368234		JN368221	JN368203	
*Polyporales*/*Fomitopsidaceae*	*Fomitopsis pinicola*	AFTOL 770	AY705967	AY854083	AY684164		AY786056
-/*Grifolaceae*	*Grifola frondosa*	AFTOL 701	AY705960	AY854084	AY629318		AY786057
*Thelephorales*/*Bankeraceae*	*Boletopsis leucomelaena*	PBM2678	DQ435797	DQ484064	DQ154112		GU187820
-/*Thelephoraceae*	*Thelephora ganbajun*	ZRL20151295	KY418962	LT716082	KY418908		KY419043

The newly generated sequences are in boldface.

## Data Availability

All sequence data generated for this study can be accessed via GenBank: https://www.ncbi.nlm.nih.gov/genbank/ (accessed on 7 July 2023).

## Supplementary Materials

The following supporting information can be downloaded at: https://www.mdpi.com/article/10.3390/jof10010022/s1, File S1: The concatenated alignment of nrSSU, ITS, nrLSU, mtSSU, and *RPB2* regions. Figure S1: Phylogenetic relationship within *Hymenochaetales,* inferred from the nrSSU region. Figure S2: Phylogenetic relationship within *Hymenochaetales,* inferred from the ITS region. Figure S3: Phylogenetic relationship within *Hymenochaetales,* inferred from the nrLSU region. Figure S4: Phylogenetic relationship within *Hymenochaetales,* inferred from the mtSSU region. Figure S5: Phylogenetic relationship within *Hymenochaetales,* inferred from the *RPB2* region.
