# Supplementary figures and images for: Umbellaceae fam. nov. (Hymenochaetales, Basidiomycota) for Umbellus sinensis gen. et sp. nov. and Three New Combinations
Source: J Fungi (Basel). 2023 Dec 28;10(1):22. doi: 10.3390/jof10010022 (PMC10817349; doi:10.3390/jof10010022)

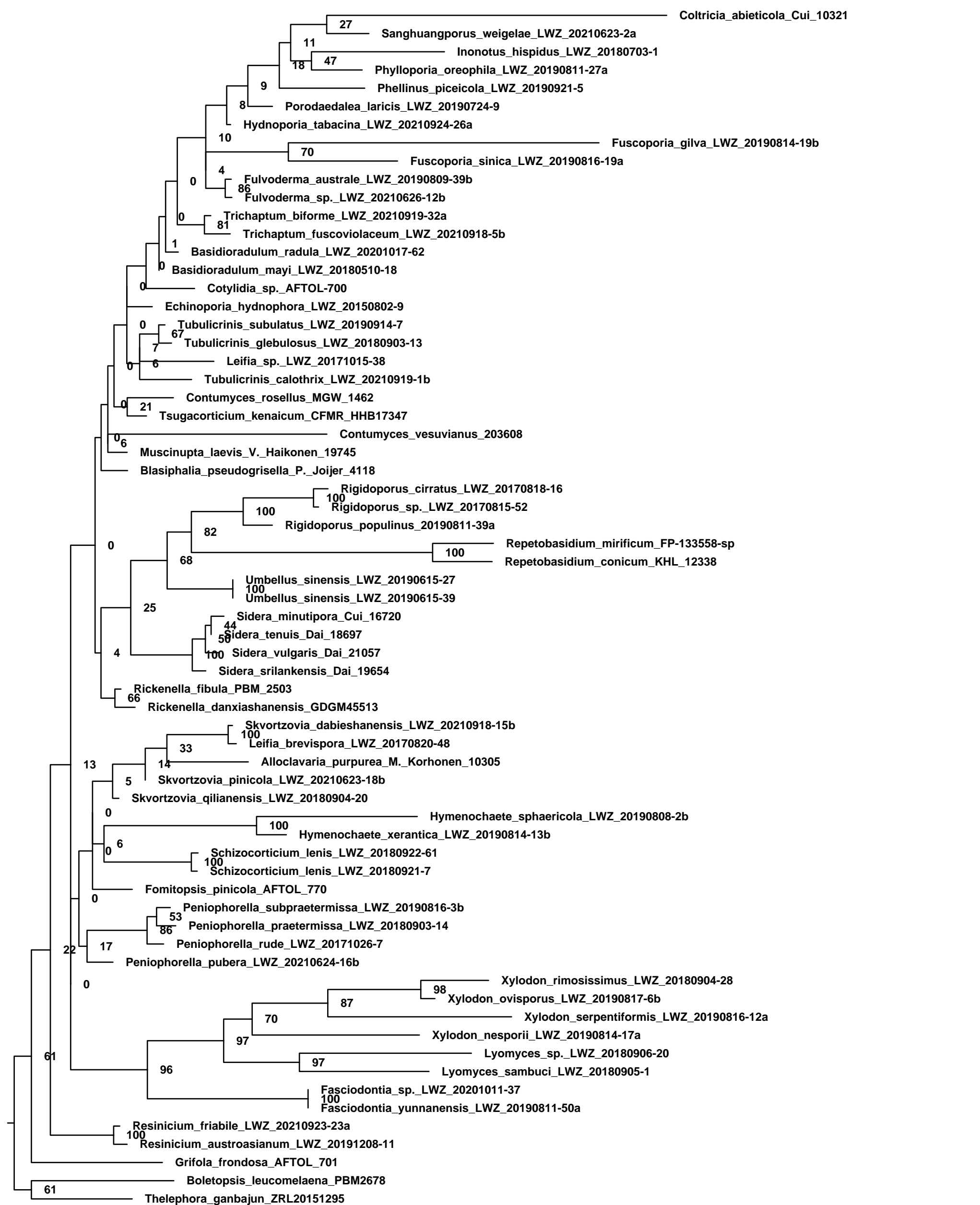

0.02

Supplement: Supplementary file 1 [file jof-10-00022-s001.zip › Figure S1 nrSSU.pdf]

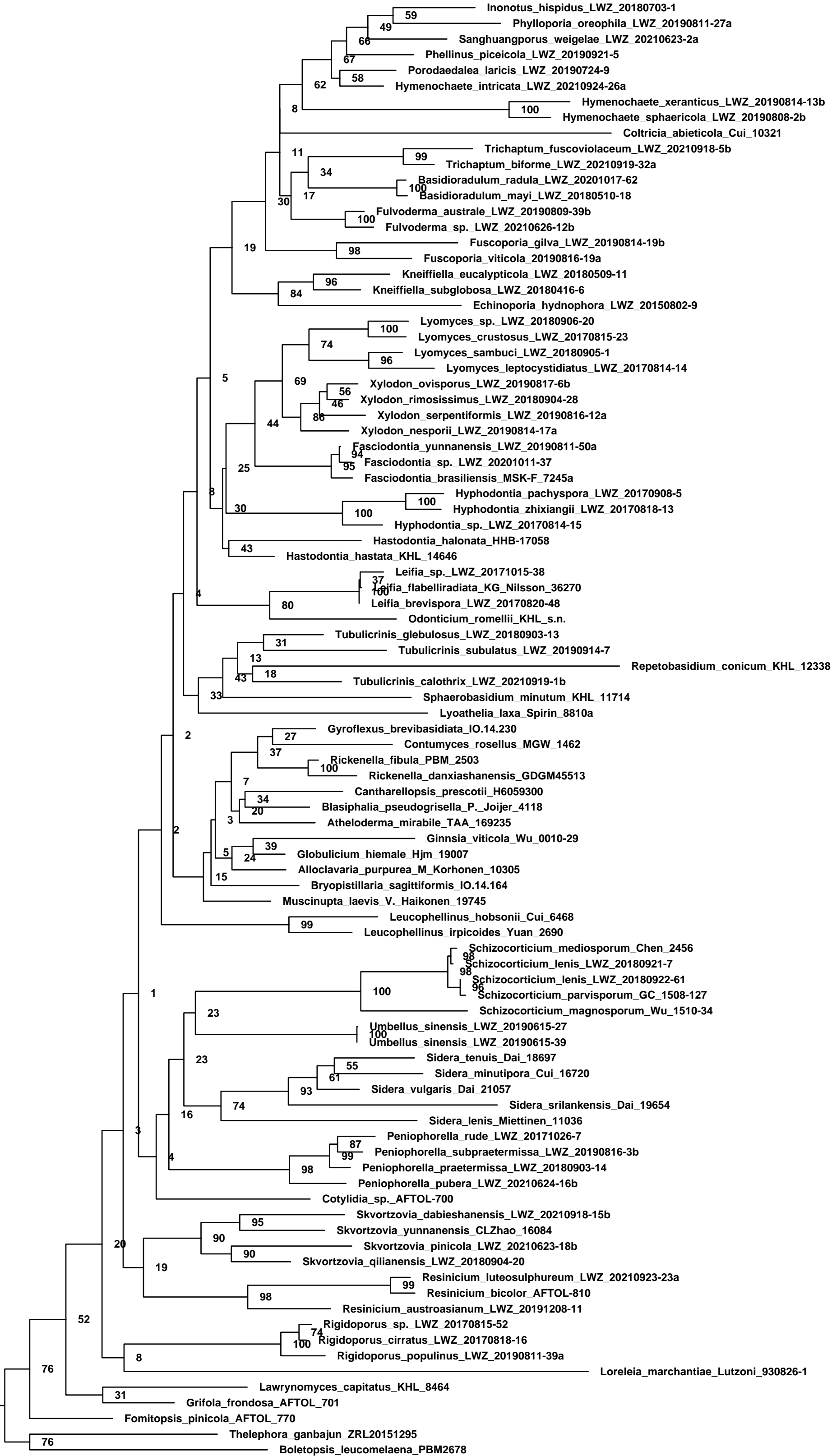

0.3

Supplement: Supplementary file 1 [file jof-10-00022-s001.zip › Figure S2 ITS.pdf]

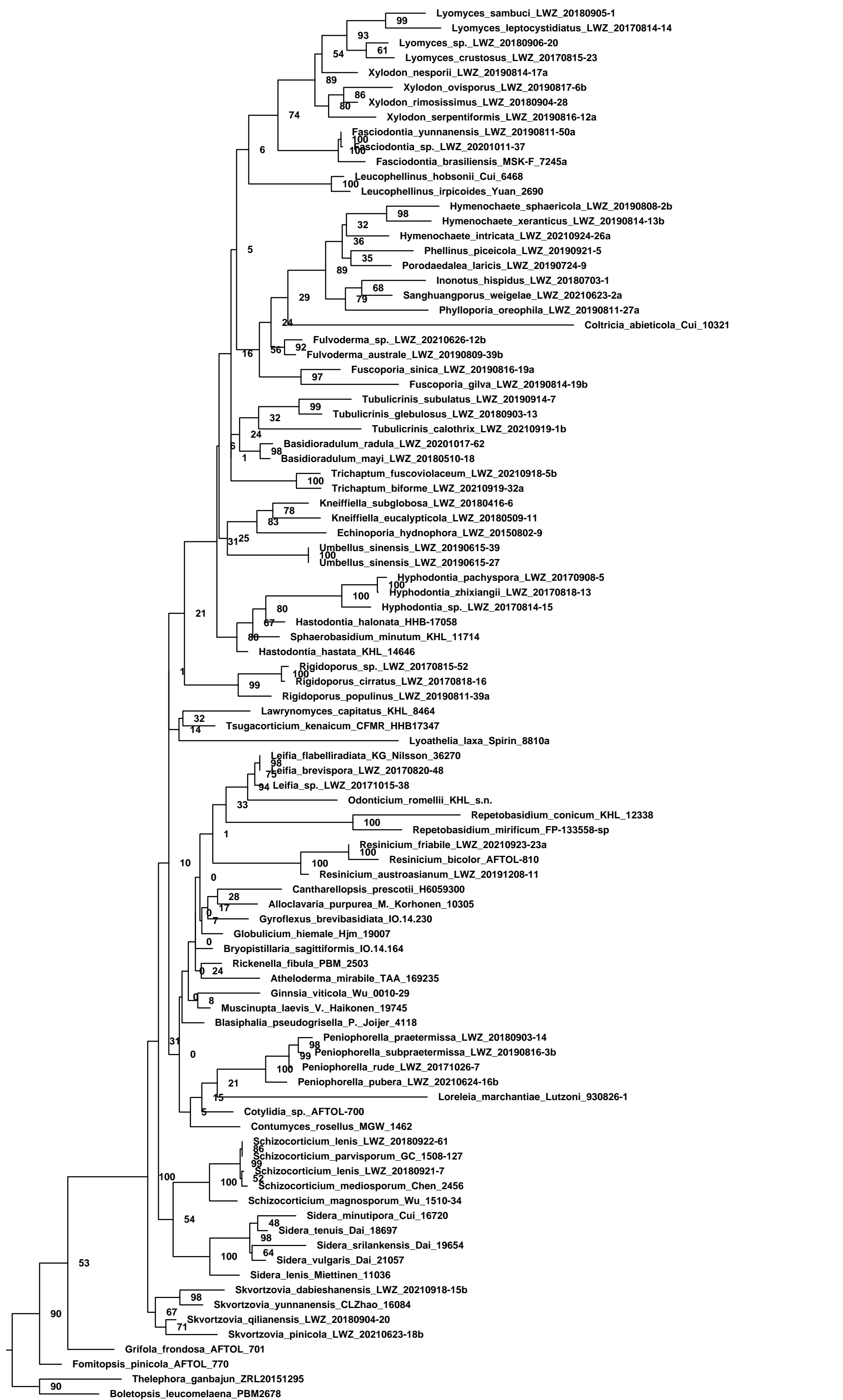

0.05

Supplement: Supplementary file 1 [file jof-10-00022-s001.zip › Figure S3 nrLSU.pdf]

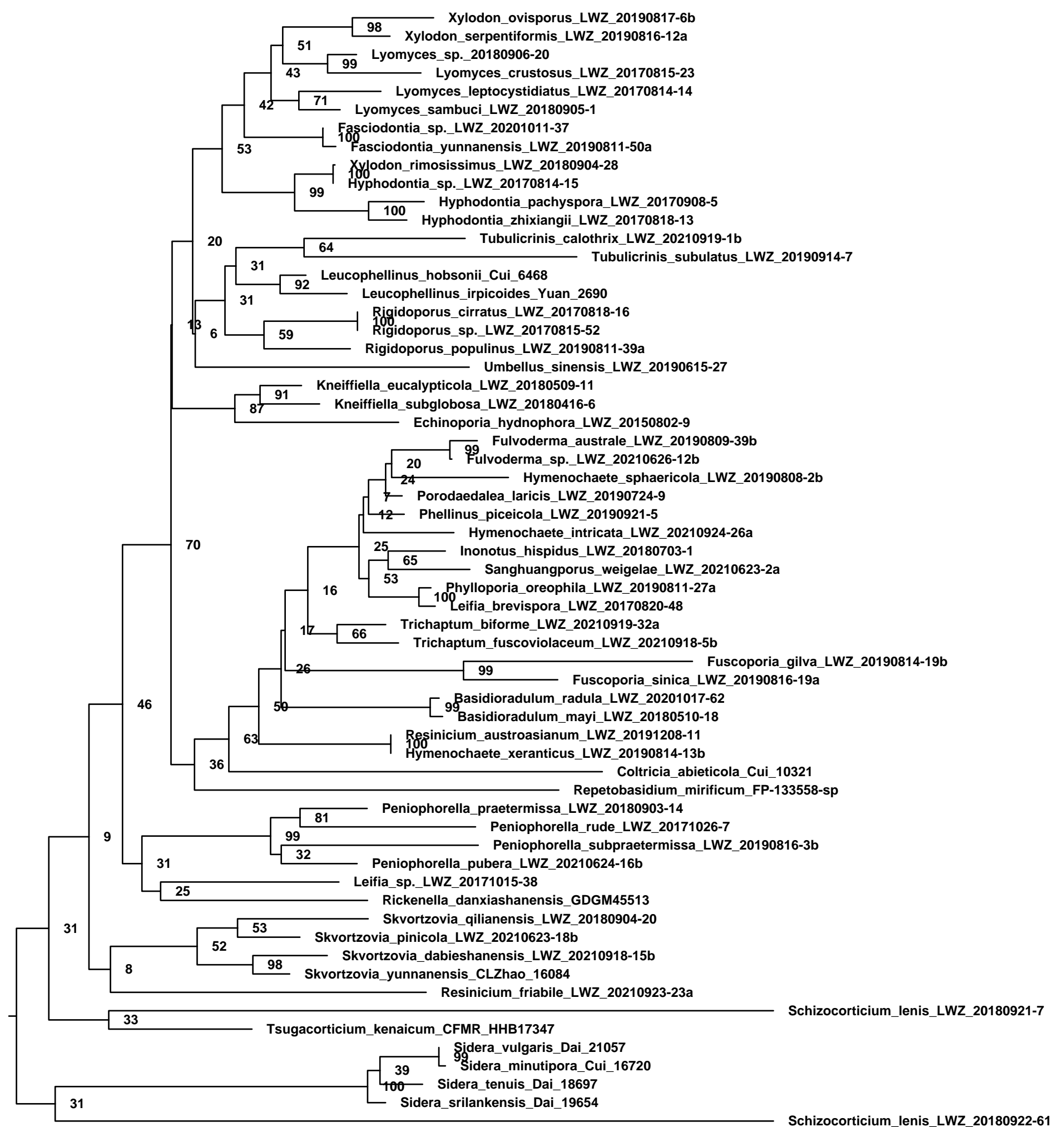

0.2

Supplement: Supplementary file 1 [file jof-10-00022-s001.zip › Figure S4 mtSSU.pdf]

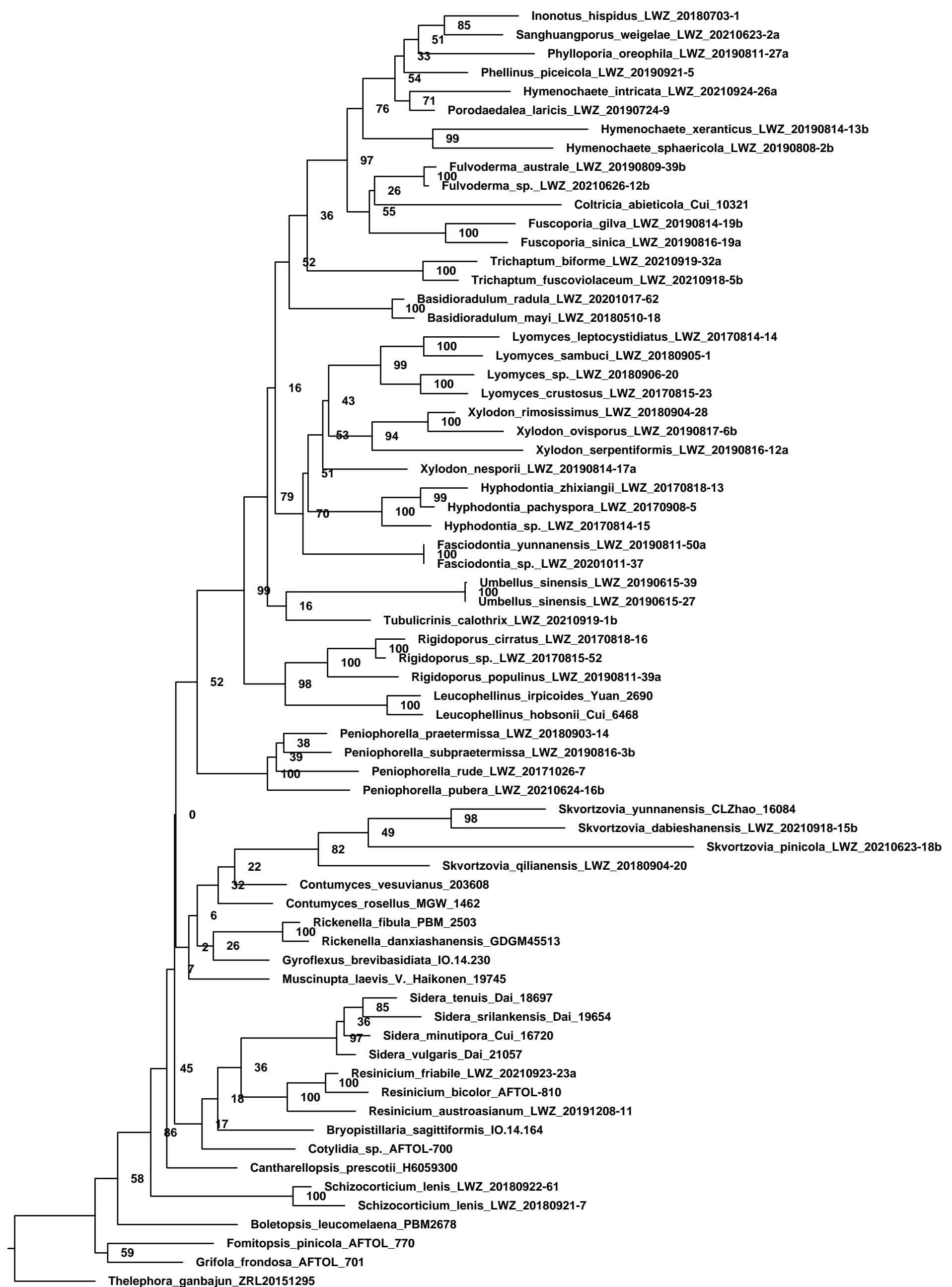

0.3

Supplement: Supplementary file 1 [file jof-10-00022-s001.zip › Figure S5 RPB2.pdf]
